# Single cell oils of the cold-adapted oleaginous yeast *Rhodotorula glacialis *DBVPG 4785

**DOI:** 10.1186/1475-2859-9-73

**Published:** 2010-09-23

**Authors:** Alberto Amaretti, Stefano Raimondi, Maurizio Sala, Lucia Roncaglia, Marzia De Lucia, Alan Leonardi, Maddalena Rossi

**Affiliations:** 1 Department of Chemistry-University of Modena and Reggio Emilia, via Campi 183, 41100 Modena, Italy

## Abstract

**Background:**

The production of microbial lipids has attracted considerable interest during the past decade since they can be successfully used to produce biodiesel by catalyzed transesterification with short chain alcohols. Certain yeast species, including several psychrophilic isolates, are oleaginous and accumulate lipids from 20 to 70% of biomass under appropriate cultivation conditions. Among them, *Rhodotorula glacialis *is a psychrophilic basidiomycetous species capable to accumulate intracellular lipids.

**Results:**

*Rhodotorula glacialis *DBVPG 4785 is an oleaginous psychrophilic yeast isolated from a glacial environment. Despite its origin, the strain abundantly grew and accumulated lipids between -3 to 20°C. The temperature did not influence the yield coefficients of both biomass and lipids production, but had positive effect on the growth rate and thus on volumetric productivity of lipid. In glucose-based media, cellular multiplication occurred first, while the lipogenic phase followed whenever the culture was limited by a nutrient other than glucose. The extent of the carbon excess had positive effects on triacylglycerols production, that was maximum with 120 g L^-1 ^glucose, in terms of lipid concentration (19 g L^-1^), lipid/biomass (68%) and lipid/glucose yields (16%). Both glucose concentration and growth temperature influenced the composition of fatty acids, whose unsaturation degree decreased when the temperature or glucose excess increased.

**Conclusions:**

This study is the first proposed biotechnological application for *Rhodotorula glacialis *species, whose oleaginous biomass accumulates high amounts of lipids within a wide range of temperatures through appropriate cultivation C:N ratio. Although *R. glacialis *DBVPG 4785 is a cold adapted yeast, lipid production occurs over a broad range of temperatures and it can be considered an interesting microorganism for the production of single cell oils.

## Background

Oleaginous microorganisms, such as yeasts, fungi, and microalgae, can accumulate high amounts of neutral storage lipids under appropriate cultivation conditions [1,2], therefore their potential as sources of triacylglycerols (TAG) has attracted considerable attention. The utilization of microbial TAG has been increasingly explored during the past decade in the biofuels industry [3]. Microbial lipids can be successfully used to produce biodiesel by catalyzed transesterification with short chain alcohols, although they have not been industrially exploited until now [4,5]. Plant oils are the major feedstock for biodiesel production but encounter limitations regarding their availability at competitive price. These drawbacks decreases the attractiveness of biodiesel as a competitive alternative to petroleum-based fuel. The microbial production of lipids promises to overcome these limitations. In fact, the exploitation of microbial systems to produce lipids from cheap carbon sources has many advantages, including short life cycle, low affection by venue, season and climate, and possibility of process scale-up [4].

Many yeast species were found to be oleaginous and accumulated TAG from 20 to 70% of biomass under appropriate cultivation conditions. They include *Cryptococcus albidus*, *Lipomyces lipofera*, *Lipomyces starkeyi*, *Rhodosporidium toruloides*, *Rhodotorula glutinis*, *Trichosporon pullulan*, and *Yarrowia lipolytica *[2,6,7]. Lipid accumulation in oleaginous yeasts and molds has been demonstrated to occur when a nutrient in the medium (e.g. the nitrogen or the phosphorus source) becomes limited and the carbon source is present in excess. Nitrogen limitation is the most efficient condition for inducing lipogenesis. During the growth phase, nitrogen is necessary for the synthesis of proteins and nucleic acids, while the carbon flux is distributed among energetic and anabolic processes yielding carbohydrates, lipids, nucleic acids and proteins. When nitrogen gets limited, the growth rate slows down and the synthesis of proteins and nucleic acids tends to cease. In non-oleaginous species, the carbon excess remains unutilized or is converted into storage polysaccharides, while, in oleaginous species, it is preferentially channeled toward lipid synthesis, leading to the accumulation of TAG within intracellular lipid bodies [6,8].

Psychrophilic yeasts are understudied sources of biodiversity, which can play a novel role in biotechnology, thus offering an alternative to conventional microorganisms. Our previous work demonstrated that several cold-adapted yeasts can be regarded as excellent lipid producers [9]. Nonetheless, neither has the lipogenesis in psychrophilic yeasts been studied, nor have culture strategies been developed in order to improve TAG production, in the perspective of a potential utilization in biofuel industry. In the present work, the lipid composition and accumulation by the cold adapted yeast *Rhodotorula glacialis *DBVPG 4785 was investigated within a wide range of temperatures and carbon/nitrogen ratios. The strain belongs to a recently described psychrophilic basidiomycetous species, containing diverse yeast isolates from glacial environments [10,11], and has been demonstrated to accumulate intracellular lipids [9]. To our knowledge, this is the unique study that investigate microbial lipids production by *R. glacialis*, with the aim to develop a novel potential alternative feedstock for biodiesel industry.

## Results and Discussion

### Growth and lipid production at different temperatures

*Rhodotorula glacialis *DBVPG 4785 was cultured batchwise at different temperatures from -3 to 25°C in a medium containing 40 g L^-1 ^glucose. This cold adapted strain, which was isolated from an alpine glacial environment, grew at zero and sub-zero temperatures, but abundant growth was observed up to 20°C, although it did not occur at 25°C. Therefore, *R. glacialis *DBVPG 4785 can be regarded as an obligate psychrophilic strain, based on the current classification [12,13].

In the range of temperature allowing growth, the microorganism always consumed the carbon source completely. On the average, 14.1 g L^-1 ^dry biomass and 4.8 g L^-1 ^lipids were obtained from 40 g L^-1 ^glucose, without any significant effect (P > 0.05) of temperature (Table 1). Thus, the acclimation to different temperature did not affect biomass/substrate (Y_X/S _= 0.35 g g^-1^), lipid/biomass (Y_L/X _= 0.34 g g^-1^), and lipid/glucose (Y_L/S _= 0.12 g g^-1^) yield coefficients (P > 0.05).

**Table 1:** Effects of the growth temperature on growth and lipid production of *R. glacialis *DBVPG 4785

T (°C)	μ (h^-1^)	Y_L/S_ (g g^-1^)	Y_L/X_ (g g^-1^)	Y_X/S_ (g g^-1^)	Q_X_ (g L^-1 ^h^-1^)	Q_L_ (g L^-1 ^h^-1^)	Biomass (g L^-1^)	Lipids (g L^-1^)
-3	0.010^d^	0.12^a^	0.35^a^	0.33^a^	0.015^e^	0.005^d^	13.3^a^	4.6^a^
0	0.019^d^	0.13^a^	0.35^a^	0.36^a^	0.023^d^	0.007^d^	14.4^a^	5.1^a^
5	0.053^c^	0.12^a^	0.32^a^	0.36^a^	0.050^c^	0.016^c^	14.3^a^	4.7^a^
10	0.071^a^	0.12^a^	0.34^a^	0.35^a^	0.077^b^	0.026^b^	14.0^a^	4.8^a^
15	0.075^a^	0.12^a^	0.32^a^	0.36^a^	0.101^a^	0.034^a^	14.3^a^	4.7^a^
20	0.068^b^	0.12^a^	0.35^a^	0.35^a^	0.075^b^	0.023^b^	14.2^a^	5.0^a^

The strain was cultured at different temperatures in GMY medium containing 40 g L^-1 ^glucose, as described in Methods section. The final concentration of biomass and lipids, the specific growth rate (μ), the biomass/glucose, lipid/glucose, and lipid/biomass yield coefficients (Y_X/S_, Y_L/S_, and Y_L/X_, respectively), and the volumetric productivities of biomass and lipids (Q_X _and Q_L_, respectively) are reported as a function of the growth temperature. The values are means of three independent experiments. Within a column, superscripts indicate statistically similar means, P > 0.05, n = 3.

As expected, the temperature markedly affected the specific growth rate (μ). The μ increased if the temperature raised, was the highest at 15°C (0.075 h^-1^), then declined at 20°C (Table 1). As a result, volumetric productivities of both biomass (Q_X_) and lipids (Q_L_) depended by the temperature and were maximum at 15°C (0.101 and 0.034 g L^-1 ^h^-1^, respectively).

### Effects of temperature on fatty acids composition

The fatty acids (FA) with chain length ranging from 14 to 18 carbons, including their saturated (SFA), mono-unsaturated (MUFA), and poly-unsaturated (PUFA) forms, dominated the FA profile of *R. glacialis *DBVPG 4785, always accounting for more than 99% (Table 2). Eighteen-carbons FA were the most abundant (69.0 to 83.1%), followed by sixteen-carbons FA (14.0 to 27.6%). Myristic acid (C14) was approximately 3% at all the temperatures (Table 2), lauric acid (C12) was always found in traces, and the FA with 20 carbons or more were negligible. The most abundant unsaturated FA were palmitoleic (C16:1Δ^9^), oleic (C18:1Δ^9^), linoleic (C18:2Δ^9,12^), and α-linolenic (C18:3Δ^9,12,15^) acids. Other unsaturated FA were negligible, with the sole exception of γ-linolenic (C18:3Δ^6,9,12^) and stearidonic acid (C18:4Δ^6,9,12,15^) which were found in low amounts at 0 and -3°C (< 0.4% in sum) and were likely produced throughΔ6 desaturase. Although stearidonic acid is known as an intermediate in the conversion of α-linolenic acid to eicosapentaenoic acid and docosahexaenoic acid [6,14], long-chain highly-unsaturated FA were absent.

**Table 2:** Effects of the growth temperature on fatty acids (FA) composition

T (°C)	Relative abundance of FA (w/w%)												UI
		
	C14	C16	C16:1	C18	C18:1	C18:2	C18:3	Total C16	Total C18	Total SFA	Total MUFA	Total PUFA	
-3	3.4^a^	14.6^bc^	1.3^a^	5.6^b^	35.6^a^	26.0^d^	13.5^a^	15.9^bc^	80.7^b^	23.6^c^	36.9^a^	39.5^c^	1.29^b^
0	2.9^a^	13.0^c^	1.0^a^	2.7^c^	25.1^c^	48.5^a^	6.9^b^	14.0^c^	83.1^a^	18.6^d^	26.1^c^	55.4^a^	1.44^a^
5	3.0^a^	15.5^b^	1.2^a^	4.7^b^	27.8^b^	43.0^b^	4.8^c^	16.7^b^	80.3^b^	23.2^c^	29.0^b^	47.8^b^	1.29^b^
10	3.2^a^	16.0^b^	1.6^a^	5.2^b^	28.6^b^	41.0^c^	4.4^c^	17.6^b^	79.2^b^	24.4^c^	30.2^b^	45.4^b^	1.26^b^
15	2.6^a^	24.6^a^	2.1^a^	5.6^b^	23.6^c^	39.4^c^	2.1^d^	26.7^a^	70.7^c^	32.8^b^	25.7^c^	41.5^c^	1.11^c^
20	3.4^a^	25.7^a^	1.9^a^	7.8^a^	20.6^d^	39.2^c^	1.4^d^	27.6^a^	69.0^c^	36.9^a^	22.5^d^	40.6^c^	1.05^c^

*R. glacialis *DBVPG 4785 was cultured at different temperatures in GMY medium containing 40 g L^-1 ^glucose. FA within the lipid extract were transformed into the corresponding methyl-esters and were analyzed as described in Methods section. The relative abundance of each FA, the sum of 16- and 18-carbons FA, the sum of the saturated, mono-unsaturated, and poly-unsaturated ones (SFA, MUFA, and PUFA, respectively), and the unsaturation index (UI) are reported as a function of the growth temperature. The values are means of three independent experiments. Within a column, superscripts indicate statistically similar means, P > 0.05, n = 3.

To regulate membrane fluidity and functionality at low temperatures, *R. glacialis *DBVPG 4785 exploited diverse changes in lipid composition, consisting in the increased degree of unsaturation, herein described by the quantitative descriptor UI (unsaturation index), and the incorporation of higher amounts of 18-carbons FA at the expenses of the 16-carbons ones (Table 2). The relative abundance of SFA diminished from 36.9 to 18.6% if the temperature decreased from 20 to 0°C, due to the decrease of both palmitic and stearic acids (P ≤ 0.05). The opposite trend was observed for both linoleic and α-linolenic acids (P ≤ 0.05), which were the major responsible for the increase of the UI (P ≤ 0.05). The production of 18-carbons FA seems to be an adaptive feature of the yeasts coming from cold environments, and may be due to the necessity to elongate FA beyond C16 in order to introduce additional double bonds by Δ12 and Δ15 desaturases [9]. The relative amount of palmitoleic acid slightly increased with temperature, but never accounted for more than 2.1%.

At -3°C, the FA profile differed from the trend described above, especially due to the diverse distribution within the C18 series. The lipid extract was richer in C18, C18:1, and C18:3, while a lower amount of C18:2 was observed. The occurrence of a remarkably high amount of C18:3 may arise from the NaCl supplementation to prevent freezing, since salinity has been demonstrated to improve membrane fluidity in certain yeasts through the incorporation of FA with higher unsaturation [15,16].

### Effects of glucose concentration on growth and lipid production

Most of oleaginous yeasts accumulate storage lipids when a nutrient becomes exhausted but the carbon source is still available and continues to be assimilated by the cells which progressively become obese [1,17,18]. To investigate whether lipid production by *R. glacialis *DBVPG 4785 can be improved through a proper nutrient limitation, the strain was cultured at 10°C with the following initial glucose concentrations: 1.6, 4, 8, 16, 40, 80, 120, and 160 g L^-1^. Since the yeast extract contained 8% (w/w) nitrogen (data not shown), the corresponding C:N ratios were 5.6, 8.5, 13, 23, 52, 101, 149, and 198, respectively. For initial glucose ranging from 1.6 to 40 g L^-1^, the exponential growth occurred with the same specific rate (μ) of 0.071 h^-1^, which decreased with higher initial glucose concentration (Table 3).

**Table 3:** Effects of glucose concentration on growth and lipid production of *R. glacialis *DBVPG 4785

Glucose (g L^-1^)	μ (h^-1^)	Y_L/S_ (g g^-1^)	Y_L/X_ (g g^-1^)	Y_X/S_ (g g^-1^)	Q_X_ (g L^-1 ^h^-1^)	Q_L_ (g L^-1 ^h^-1^)	Cells (ml^-1^)	Biomass (g L^-1^)	Carbohydrates (g L^-1^)	Lipids (g L^-1^)
1.6	0.070^a^	0.07^d^	0.06^e^	1.31^a^	0.045^e^	0.0030^f^	1.2e+08^c^	2.1^h^	0.1^f^	0.12^g^
4	0.071^a^	0.05^d^	0.05^e^	0.94^b^	0.052^d^	0.0026^f^	3.5e+08^b^	3.8^g^	0.3^e^	0.19^g^
8	0.070^a^	0.05^d^	0.09^e^	0.61^c^	0.052^d^	0.0044^e^	4.0e+08^ab^	4.9^f^	0.8^d^	0.42^f^
16	0.072^a^	0.09^c^	0.20^d^	0.44^d^	0.056^c^	0.011^d^	4.2e+08^a^	7.0^e^	1.6^c^	1.36^e^
40	0.072^a^	0.12^b^	0.34^c^	0.35^e^	0.075^b^	0.025^c^	4.5e+08^a^	14^d^	3.7^b^	4.74^d^
80	0.065^b^	0.15^a^	0.55^b^	0.28^f^	0.076^b^	0.042^b^	4.4e+08^a^	22^c^	5.4^a^	12^c^
120	0.060^bc^	0.16^a^	0.68^ba^	0.23^g^	0.079^a^	0.054^a^	4.3e+08^a^	28^b^	4.8^a^	19^b^
160	0.057^c^	0.16^a^	0.68^ba^	0.23^g^	0.080^a^	0.054^a^	4.4e+08^a^	33^a^	5.0^a^	22^a^

The strain was cultured at 10°C in GMY medium containing different glucose concentration, as described in Methods section. The final concentration of biomass, cell-associated carbohydrates, and lipids, the specific growth rate (μ), the biomass/glucose, lipid/glucose, and lipid/biomass yield coefficients (Y_X/S_, Y_L/S_, and Y_L/X_, respectively), and the volumetric productivities of biomass and lipids (Q_X _and Q_L_, respectively) are reported as a function of initial glucose concentration. The values are means of three independent experiments. Within a column, superscripts indicate statistically similar means, P > 0.05, n = 3.

In the range between 1.6 and 8 g L^-1 ^glucose, the culture was carbon limited and entered into stationary phase in correspondence with glucose exhaustion. Intracellular lipids were scarce, accounting for less than 10% of biomass (Table 3).

**Figure 1:**
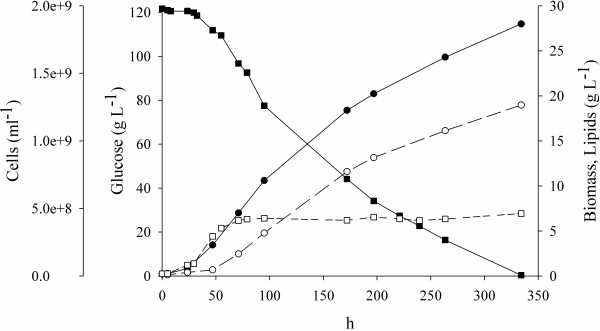
**Timecourse of a batch culture of *Rhodotorula glacialis *DBVPG 4785**. The strain was cultured at 10°C in GMY medium containing 120 g L^-1 ^glucose. In these conditions, growth and lipid production occurred through two stages. The multiplication of cells took place first and finished when a nutrient other than the carbon source was exhausted, while lipid production occurred mostly afterwards. The black square, black circle, white square, and white circle represent glucose, biomass, cells counts, and lipids, respectively.

Nutrients were nearly balanced with 16 g L^-1 ^glucose, whereas the culture was limited by a nutrient other than the carbon source at higher concentrations. In these conditions growth and lipid production occurred through two stages (Fig. 1). At the end of the growth phase (72 h), approximately 16 g L^-1 ^glucose were consumed and lipids were the 20% of biomass. Lipid production occurred mostly afterwards, consuming the glucose still available at the rate of 0.34 g L^-1 ^h^-1 ^without any relation to the initial concentration, even though the rate gradually decreased. The carbon source was always exhausted for initial glucose concentrations up to 120 g L^-1^. Cells did not multiply during the lipogenic phase, while biomass dry weight augmented in proportion to glucose consumption because of the accumulation of intracellular lipids (Fig. 1). The highest rate of lipid production was 0.080 g L^-1 ^h^-1^, and gradually decreased along with the decrease of the glucose consumption rate.

With the increase of initial glucose concentration, final cell counts did not increase above 4.5e+08 ml^-1^, while a more efficient conversion of glucose into lipids was achieved (Table 3). With 120 and 160 g L^-1 ^glucose, the highest lipid production in terms of lipid concentration (19 and 22 g L^-1^, respectively), lipid content of biomass (68%), lipid/glucose yield coefficient (16%), and productivity (0.054 g L^-1 ^h^-1^) was attained.

The lipid extract from a culture grown on 120 g L^-1 ^glucose was subjected to ^1^H-NMR spectroscopy. The chemical shifts of all the signals were fitted to the spectra of TAG. In particular, the triplet pattern occurring in the spectrum region ranging from 2.20 and 2.45 ppm provided evidence confirming the absence of free FA [19].

The data herein reported confirmed that the extent of the carbon excess had largely positive effects on lipid production by *R. glacialis *DBVPG 4785. Cell-associated carbohydrates were also positively affected by glucose. However, they became less abundant than lipids with the increase of glucose excess (Table 3), since the metabolism was preferentially channeled toward the accumulation of neutral lipids.

### Effects of glucose concentration on fatty acids composition

*R. glacialis *DBVPG 4785 produced higher amounts of PUFA (e.g. C18:3) at the expenses of MUFA (e.g. C18:1) in carbon limited cultures compared with carbon excess conditions (P ≤ 0.05) (Table 4). Conversely, an excess of glucose increased MUFA and reduced PUFA.

**Table 4:** Effects of glucose concentration on fatty acids (FA) composition

Glucose (g L^-1^)	Relative abundance of FA (w/w %)												UI
		
	C14	C16	C16:1	C18	C18:1	C18:2	C18:3	Total C16	Total C18	Total SFA	Total MUFA	Total PUFA	
1.6	3.1^a^	14.4^a^	0.4^a^	7.4^a^	4.3^e^	43.8^ab^	26.6^a^	14.8^a^	82.1^a^	25.3^a^	4.3^f^	70.4^a^	1.72^a^
4	3.1^a^	15.1^a^	1.1^a^	7.2^a^	6.3^de^	43.1^ab^	24.1^a^	16.2^a^	80.7^a^	25.4^a^	7.4^e^	67.2^a^	1.66^a^
8	2.6^a^	13.2^a^	1.4^a^	6.8^a^	7.7^d^	43.5^ab^	24.8^a^	14.6^a^	83.1^a^	22.6^a^	9.1^e^	68.3^a^	1.71^a^
16	3.4^a^	16.6^a^	1.6^a^	6.6^a^	20.3^c^	46.7^a^	4.8^b^	18.2^a^	78.5^a^	26.6^a^	21.9^d^	51.5^b^	1.30^b^
40	3.2^a^	16.0^a^	1.6^a^	5.2^a^	28.6^b^	41.0^b^	4.4^b^	17.6^a^	79.0^a^	24.4^a^	30.2^c^	45.4^c^	1.26^bc^
80	3.1^a^	14.2^a^	1.2^a^	6.3^a^	33.4^a^	36.2^c^	5.5^b^	15.4^a^	81.5^a^	23.6^a^	34.6^bc^	41.7^d^	1.24^c^
120	4.6^a^	14.4^a^	0.5^a^	6.3^a^	36.3^a^	33.1^c^	4.8^b^	15.0^a^	80.4^a^	25.3^a^	36.8^ab^	37.9^e^	1.17^d^
160	4.4^a^	14.0^a^	1.5^a^	6.3^a^	35.5^a^	34.6^c^	3.8^b^	15.4^a^	80.1^a^	24.7^a^	37^a^	38.4^e^	1.18^d^

*R. glacialis *DBVPG 4785 was cultured at different temperatures in GMY medium containing 40 g L^-1 ^glucose. FA within the lipid extract were transformed into the corresponding methyl-esters and were analyzed as described in Methods section. The relative abundance of each FA, the sum of 16- and 18-carbons FA, the sum of the saturated, mono-unsaturated, and poly-unsaturated ones (SFA, MUFA, and PUFA, respectively), and the unsaturation index (UI) are reported as a function initial glucose concentration. The values are means of three independent experiments. Within a column, superscripts indicate statistically similar means, P > 0.05, n = 3.

In carbon limited cultures, the initial concentration of glucose did not influence the relative abundance of C18:3, C18:2, and C18:1. With the increase of the glucose concentration up to 16 g L^-1 ^and more, the mean amount of C18:3 became much lower, the amount of C18:1 progressively augmented, and C18:2 diminished. As a consequence, the UI was higher (P ≤ 0.05) under carbon limitation than in presence of glucose excess and further decreased with the increase of glucose concentration (Table 4).

These data corroborate the observation that in oleaginous yeasts and fungi the proportion of PUFA increases when nitrogen is in excess, while the rate of the desaturation reaction becomes low and the saturated fatty acids are not converted to PUFA with high carbon/nitrogen ratios [20,21].

## Conclusions

This study explored the production of intracellular lipids by the psychrophilic oleaginous yeast *Rhodotorula glacialis *DBVPG 4785 as a function of the growth temperature and the C:N ratio of the medium, with the aim to determine whether this strain could be exploited for biodiesel production. The strain is oleaginous and accumulates high amounts of lipids within the biomass when it is cultured in a medium with high C:N ratio. The fatty acid compositional profile and the content of neutral lipids are quite similar to those of soybean oil and rapeseed oil, indicating that lipids produced by *R. glacialis *DBVPG 4785 have potential as a feedstock for biodiesel production.

Therefore, this yeast could be considered an intriguing microorganism for the production of single cell oils, since it can grow and produce lipids over a wide range of temperatures, although it originates from a cold habitat. Furthermore, the present study is the first proposed biotechnological application for *Rhodotorula glacialis *species, whose oleaginous biomass accumulates lipids through appropriate cultivation conditions up to 69% and may find application as novel feedstock for biodiesel production.

## Methods

### Strain and culture conditions

*Rhodotorula glacialis *DBVPG 4785 was obtained from the Industrial Yeasts Collection DBVPG (University of Perugia, Italy, http://www.agr.unipg.it/dbvpg. The strain was aerobically cultured in GMY medium, which contained 8 g L^-1 ^KH_2_PO_4_, 0.5 g L^-1 ^MgSO_4 _7H_2_O, and 3 g L^-1 ^yeast extract (Difco Laboratories, Sparks, MD, USA), final pH 5.5 [22]. Glucose was autoclaved separately and supplied at 40 g L^-1^, unless otherwise stated.

### Growth experiments and bioreactor operation

Different glucose concentrations and different temperatures were tried out in bioreactor batch cultures using a Labfors apparatus (Infors AG, Bottmingen, Switzerland) with 2 L working volume. *R. glacialis *DBVPG 4785 was cultured at 10°C in GMY medium containing 1.6, 4, 8, 16, 40, 80, 120, and 160 g L^-1 ^glucose, or at -3, 0, 5, 10, 15, and 20°C with 40 g L^-1 ^glucose. At -3°C, 30 g L^-1 ^NaCl were supplied to prevent freezing. In any case, the bioreactor was inoculated (10% v/v) with a 48-h seed culture grown at the target temperature on 4 g L^-1 ^glucose. The culture was stirred at 400 rpm and sparged with 0.5 v/v/min filter-sterilized air. Samples were collected periodically to monitor the turbidity at 600 nm and to determine the concentration of cells, biomass, glucose, and lipids.

The cells were counted in a Bürker chamber and biomass dry weight was determined gravimetrically using pre-weighed cellulose nitrate membrane filters. Total cellular carbohydrates were quantified using anthrone reagent [23]. Glucose was analyzed by HPLC equipped with refractive index detector (HPLC System, 1200 Series, Agilent Technologies, Santa Clara, CA). The analysis was performed with an Aminex HPX-87 H ion exclusion column and 0.005 M H_2_SO_4 _(0.6 ml min^-1^) as the mobile phase.

### Calculation of the specific growth rates, yield coefficients and volumetric productivities

The specific growth rate (μ) was calculated using turbidity values in the exponential phase of the growth curve. The weights of biomass, lipids, and consumed glucose were used to calculate biomass/glucose, lipid/glucose, and lipid/biomass yield coefficients (Y_X/S_, Y_L/S_, and Y_L/X_, respectively), expressed as g g^-1^. The volumetric productivities of biomass and lipids (Q_X _and Q_L_, respectively) were calculated by dividing their concentrations with the corresponding culture time.

### Lipid analysis

Biomass from 50 ml culture samples was harvested, washed with distilled water, frozen at -80°C and lyophilized. Lipids were extracted from 1 g of lyophilized biomass extracted with 50 ml chloroform:methanol mixture [11]. Solvents were removed and lipids were determined gravimetrically. To determine the relative composition of fatty acids (FA), the lipids were subjected to methanolysis and the fatty acyl methyl esters were analyzed by GC-MS [11,24]. Quadrupole GC-MS system (HP5890 Series II gas chromatograph-HP5972 mass selective detector) equipped with HP-5 capillary column (Agilent Technologies) and EI ionisation detector (70 eV) was used. The injection temperature was 280°C and oven temperature was programmed from 80°C (1 min isotherm) to 130°C at a rate of 50°C min^-1^, then to 280°C at a rate of 5°C min^-1 ^(20 min isotherm at 280°C). The unsaturation index (UI) was calculated as the number of the double bonds of fatty acids multiplied by their relative amount.

### Statistical analysis

All values are means of three separate experiments. Differences in means among the growth temperatures were analyzed using two-way ANOVA with repeated measures with the group as the first factor and temperature as the second factor, followed by Bonferroni *post hoc *comparisons. Differences were considered statistically significant for P ≤ 0.05.

## Competing interests

The authors declare that they have no competing interests.

## Authors' contributions

AA and SR conceived the study, performed the experimental work and drafted the manuscript. MS and LR performed lipid extraction and GC-MS analysis. MDL contributed to the fermentation experiments and chemical analysis. AL accomplished data interpretation and statistical analysis. MR participated in the design of the study and writing of the manuscript. All authors read and approved the final manuscript.
